# Aphid infestations reduce monarch butterfly colonization, herbivory, and growth on ornamental milkweed

**DOI:** 10.1371/journal.pone.0288407

**Published:** 2023-07-26

**Authors:** Bernadette M. Mach, William Long, Jaret C. Daniels, Adam G. Dale

**Affiliations:** 1 Entomology and Nematology Department, Institute of Food and Agricultural Sciences, University of Florida, Gainesville, FL, United States of America; 2 Florida Museum of Natural History, Gainesville, FL, United States of America; ICAR-Central Insitute for Cotton Research, INDIA

## Abstract

Anthropogenic disturbance is driving global biodiversity loss, including the monarch butterfly (*Danaus plexippus*), a dietary specialist of milkweed. In response, ornamental milkweed plantings are increasingly common in urbanized landscapes, and recent evidence indicates they have conservation value for monarch butterflies. Unfortunately, sap-feeding insect herbivores, including the oleander aphid (*Aphis nerii*), frequently reach high densities on plants in nursery settings and urbanized landscapes. Aphid-infested milkweed may inhibit monarch conservation efforts by reducing host plant quality and inducing plant defenses. To test this, we evaluated the effects of oleander aphid infestation on monarch oviposition, larval performance, and plant traits using tropical milkweed (*Asclepias curassavica*), the most common commercially available milkweed species in the southern U.S. We quantified monarch oviposition preference, larval herbivory, larval weight, and plant characteristics on aphid-free and aphid-infested milkweed. Monarch butterflies deposited three times more eggs on aphid-free versus aphid-infested milkweed. Similarly, larvae fed aphid-free milkweed consumed and weighed twice as much as larvae fed aphid-infested milkweed. Aphid-free milkweed had higher total dry leaf biomass and nitrogen content than aphid-infested milkweed. Our results indicate that oleander aphid infestations can have indirect negative impacts on urban monarch conservation efforts and highlight the need for effective Lepidoptera-friendly integrated pest management tactics for ornamental plants.

## Introduction

Anthropogenic disturbance is driving global declines in biodiversity across many taxa, including insects [1–3]. Rapid urbanization is a leading form of anthropogenic disturbance [4, 5], where natural and agricultural lands are rapidly replaced with impervious surfaces and ornamental plants. Urban and residential development dramatically affects many insect taxa, both positively and negatively, because of their highly modified plant assemblages, typical dominance by nonnative and cultivated plant species, higher fertilizer and pesticide inputs, more frequent disturbance, and fragmented habitable areas compared to surrounding natural areas [6, 7]. Although aesthetic enhancement, economic value, and human use functionality are at the forefront of urban landscape design and maintenance decisions, the public is increasingly aware of biodiversity challenges and urban landscapes have become hotspots for wildlife conservation efforts with strong potential added value [8–11].

Recent evidence indicates that urban landscapes can indeed support diverse insect populations if designed and managed appropriately [10, 12–15]. Initiatives such as the National Pollinator Garden Network’s Million Pollinator Garden Challenge™ [16], Monarch Watch’s Monarch Waystation Program [17], and the Pollinator Partnership’s Bee Friendly Garden program [18] all include guidance on creating insect conservation habitat in urban landscapes. However, insect conservation efforts in urban landscapes can be hindered due to high pest pressure [7] and the indiscriminate widespread use of non-native ornamental plants [14, 15]. These challenges may be further complicated when considering conservation of lepidopterans, a popular choice for the public given their charismatic appearance and role as pollinators, which include taxa historically regarded as plant pests as well as species of conservation concern.

Monarch butterfly (*Danaus plexippus*, L.) populations have declined by over 80% in recent decades primarily due to habitat loss [19–22], leading to the recent listing of monarchs as “Endangered” by the International Union for Conservation of Nature [23]. Monarch larvae are dietary specialists, feeding exclusively on milkweed and close relatives (family Apocynaceae, subfamily Asclepiadoideae). Thus, monarch conservation efforts largely focus on planting milkweed to enhance breeding habitat. Tropical milkweed (*Asclepias curassavica*, L.) is a species whose use outside of its native range in southern Mexico, Central America, and South America has received widespread criticism. This criticism is due in part to evidence that it can disrupt monarch migration through the U.S. by shifting diapause [24] or encouraging residency outside of their typical overwintering grounds in southern Mexico [25]. In addition, tropical milkweed has elevated cardenolide concentrations compared to other milkweed species [26–29] and recent evidence indicates tropical milkweed may become a less suitable, more toxic host under projected climate warming [26]. Tropical milkweed can also increase pathogen exposure to monarchs by supporting prolonged, high-density residency [30, 31]. Despite these well-documented concerns, tropical milkweed remains by far the most commercially available milkweed species produced in nurseries and planted in urbanized landscapes throughout southern North America due to its aesthetic appeal and ease of propagation. Increased plant availability and consumer engagement with conservation have spurred a multitude of organized efforts and initiatives to create monarch conservation habitats in urban gardens, parks, and schools throughout North America [17, 32–34].

Due to multiple anthropogenic factors, many sap-feeding insects tend to reach high densities on plants in urbanized landscapes [7]. The oleander aphid (*Aphis nerii*, Fonscolombe, 1841) is no exception [35] and has become a pervasive pest of milkweed and other Apocynaceae taxa in urban gardens throughout North America [36]. High density oleander aphid infestations of milkweed cause leaf chlorosis, leaf senescence, honeydew and sooty mold accumulation, and even plant death, which may have under-recognized negative impacts on monarch conservation efforts. Elevated sap-feeding herbivore densities can also reduce the fitness of herbivores feeding on the same host by reducing host plant nutritive quality or increasing the efficacy of constitutive defenses [37]. Cardenolides are cardiac glycosides that function as secondary defensive chemicals in milkweed and are highest in tropical milkweed compared to its native congeners [38]. Previous work using two native milkweed species demonstrated that oleander aphids and monarchs interact via plant-mediated defensive responses, but that aphid infestations had a positive or neutral effect on monarch larval development, depending on milkweed species [39]. Tropical milkweed infested with oleander aphids exhibits density-dependent cardenolide fluctuations, reducing cardenolides at low aphid densities and increasing them at high densities [40]. Although monarchs sequester cardenolides to defend themselves [41–45], larvae experience significant trade-offs between high cardenolide protection and survival [26, 45, 46]. Given that tropical milkweed contains much higher cardenolide levels than its native congeners [38] and commonly experiences severe oleander aphid infestations in urbanized landscapes, aphids may reduce monarch colonization and fitness by reducing milkweed plant quality or altering plant defenses [40]. The objective of this study was to determine the effects of aphid infestations on monarch oviposition and larval success on tropical milkweed. We hypothesized that oleander aphid infestations would negatively affect both.

## Materials and methods

### Study system

Insecticide-free tropical milkweed were purchased and maintained in 1-gallon containers (Green Isle Gardens; Groveland, FL) for use in all experiments. Different plant cohorts were used for each component of the study. Eighteen plants were used for the oviposition study (2 treatments: aphid-free and aphid-infested, × 9 replicates), thirty plants for the larval feeding study (3 treatments: aphid-free, aphid-infested “dirty” leaves, aphid-infested “cleaned” leaves, × 10 plants per treatment), and twenty plants for the milkweed characteristics study (2 treatments: aphid-free and aphid-infested, × 10 replicates). Plants were maintained in a nursery setting at the University of Florida under natural light and temperature conditions. Plants received natural rainfall and were irrigated using drip irrigation for 10 minutes daily to avoid water stress. Monarch eggs were obtained from a local butterfly farm and field-sourced lab-maintained colony. Eggs were stored at 28°C and 75% relative humidity with a 12:12 L:D diurnal cycle until eclosion. Neonate larvae were fed fresh, herbivore-free tropical milkweed before use in our experiments. Plants assigned to aphid-free treatments were kept aphid-free using mesh cages (0.6m x 0.6 m x 0.9m) and an initial insecticidal soap (Safer®) application. Insecticidal soap is a low-impact alternative to traditional insecticides and does not leave insecticide residues that may harm monarch eggs or larvae. Plants assigned to aphid-infested treatments were seeded with 10 adult oleander aphids, and experiments were conducted approximately 2 weeks later once aphid densities were about 50 aphids per terminal growth point.

### Oviposition study

Adult monarchs were obtained from lab-reared colonies and were 2–5 days old at the start of the oviposition experiments. One adult male and one adult female were placed in a mesh cage (0.6m x 0.6m x 0.9m) with one aphid-free and one aphid-infested tropical milkweed plant (both ca. 60 cm tall) placed in opposite corners with 30 ml honey water (20% v/v) between them. Importantly, milkweed used in each cage were size matched to control for size differences that may have influenced oviposition preference. Monarchs were allowed to oviposit freely for seven days. Honey water was replenished as needed and the locations of each plant within the cage were swapped on day four. Monarch eggs take approximately four days to hatch, therefore all eggs on each plant were removed and counted on days three and seven to accurately assess total eggs laid. We conducted this experiment twice in 2021 for a total of nine replicates. Each of the nine replicates used a separate pair of aphid-free and aphid-infested tropical milkweed along with a different pair of male and female adult monarchs.

### Larval feeding study

We used third instar monarch larvae for all larval feeding experiments to minimize handling stress and injury given the delicacy of earlier instars. Third instar monarch larvae were randomly assigned to one of three treatments: 1) Leaves from aphid-free milkweed, 2) leaves with sooty mold, aphids, and honeydew from aphid-infested milkweed (i.e., “dirty” leaves), and 3) leaves that had been wiped clean with water to remove sooty mold, aphids, and honeydew from aphid-infested milkweed (i.e., “cleaned” leaves). The “cleaned” leaves treatment was added to separate the effects of the buildup of honeydew, sooty mold, and shed aphid exoskeletons from the induced plant defense effects of oleander aphid feeding alone. Each larva was placed in a Petri dish (100mm x 15mm) with one leaf and stored at 28°C and 75% relative humidity with a 12:12 L:D diurnal cycle for seven days. A single new milkweed leaf was added to each Petri dish when >50% of the old leaf was consumed or every 48 h, whichever came first. Leaves were sourced from each of 10 milkweed plants per treatment, ensuring that no single plant had more than one leaf removed per day. Total leaf area consumed (cm^2^) per larva was calculated using LeafByte [47]. Final instar attained and larval weight were measured after seven days. Although using excised leaves excludes some potential plant-mediated effects of aphid infestation on milkweed, namely reduced latex pressure that may benefit larval feeding [48], conducting the experiment this way allowed us to control for several factors such as light exposure, microclimate, and environmental disturbances. Additionally, tropical milkweed has minimal latex exudation compared to other milkweed species so we did not anticipate that the lack of latex exudation in excised leaves had any significant impact on monarch herbivory [49]. We conducted this experiment once in June 2021 (n = 27).

### Milkweed characteristics

To determine the effects of aphid density on milkweed quality and constitutive defense, ten plants were assigned to either the aphid-free treatment or aphid-infested treatment and were infested and maintained as in previous experiments for two weeks. We measured latex production by cutting the tip of the youngest fully expanded leaf and collecting exuding latex on a pre-weighed 3 cm diameter filter paper disc as in Agrawal et al. [50]. After 30 s, the filter paper was weighed, and the difference was recorded as the latex weight. As a measure of biomass, dry leaf weight was calculated by removing all fully expanded leaves from each plant, drying at 50°C for 72 h, and then weighing the remaining material. Due to low total dry leaf mass, we were unable to analyze nutrients from each plant and instead pooled 2 g of dry leaf matter from each plant for a total of 10 g dry leaf matter per aphid density treatment. For leaf nitrogen and crude protein analysis, samples were digested as in Gallaher et al. [51]. Digestate nitrogen was determined by semiautomated colorimetry [52]. Digestions were conducted at the UF Forage Evaluation Support Lab and analyses of digestate were done at the UF Ruminant Nutrition Lab. Crude protein was calculated via the Kjeldahl method as N multiplied by 6.25 [CITES 53].

### Statistical analysis

Statistical analyses were conducted using R ver. 4.1.0 [54]. Oviposition results were pooled from the two experimental replicates and analyzed using a generalized linear mixed-effects model with a Poisson distribution since eggs deposited is count data, with experimental replicate as a random effect. Larval feeding study results were analyzed using one-way Analysis of Variance (ANOVA) with leaf treatment (aphid-free, aphid-infested “dirty”, aphid-infested “cleaned”) as the independent variable and either total leaf area consumed or final larval weight as dependent variables. Results were considered significant if P < 0.05. Wet latex weight was compared between treatments using a Wilcoxon rank-sum test due to non-normal distribution of data residuals. Leaf dry weight per aphid density treatment was compared between aphid density treatments using Welch’s two sample t-test.

## Results

### Oviposition study

**Fig 1:**
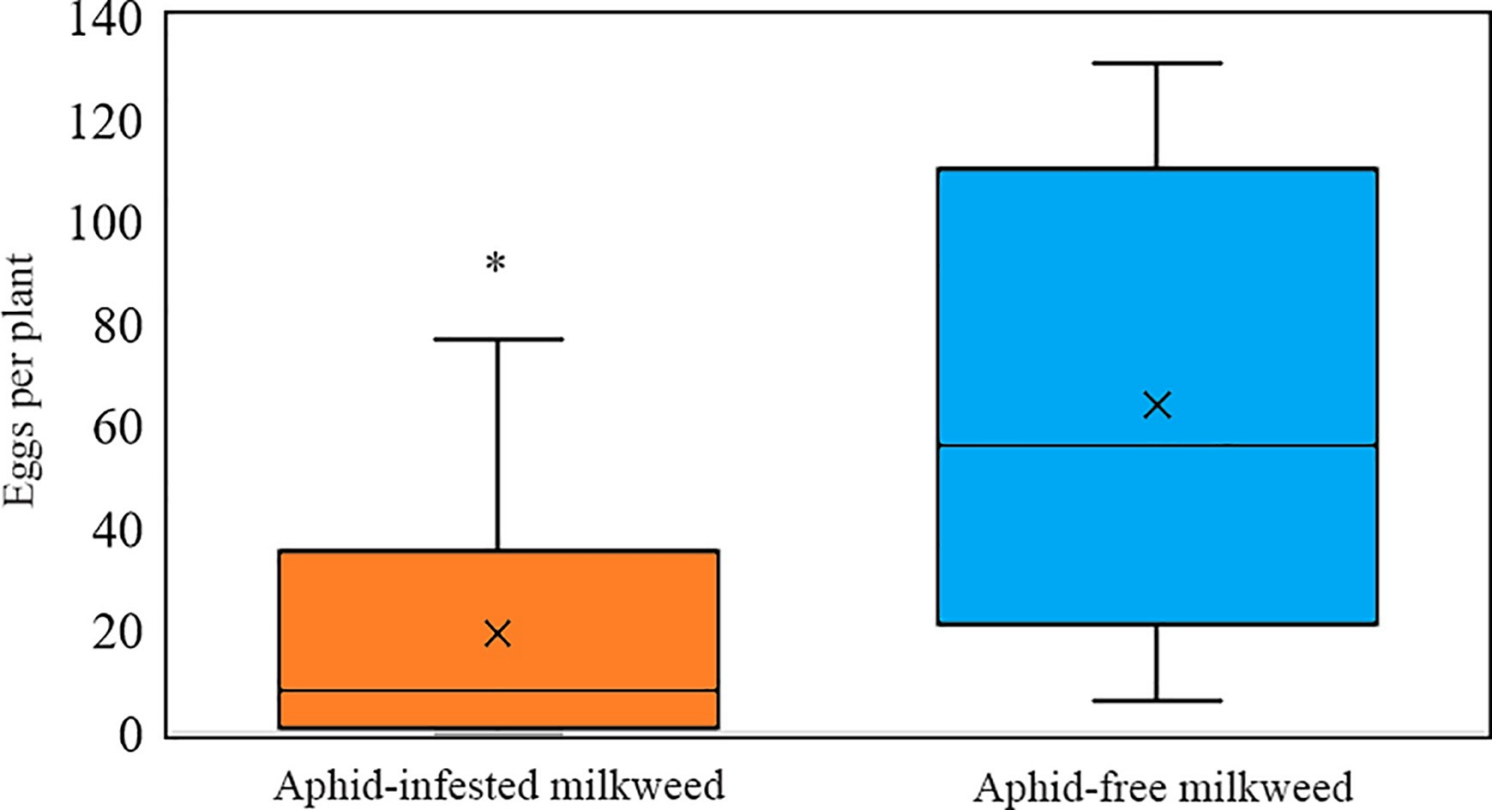
Mean (± SE) eggs deposited per plant by monarch butterflies when presented with an aphid-free and an aphid-infested tropical milkweed plant (least-squares means, Z = 13.86, P < 0.001).

Despite high variability in the number of eggs deposited per caged monarch pair, adult monarchs on average oviposited over three times as many eggs on aphid-free than aphid-infested milkweed plants (Fig 1, Z = 13.86, P < 0.001). The mean (±SE) number of eggs per aphid-free milkweed was 64 (±15.6), with a range of 6–131 eggs, whereas the mean number of eggs per aphid-infested milkweed was 19 (±8.8), with a range of 0–77 eggs.

### Larval feeding study

**Fig 2:**
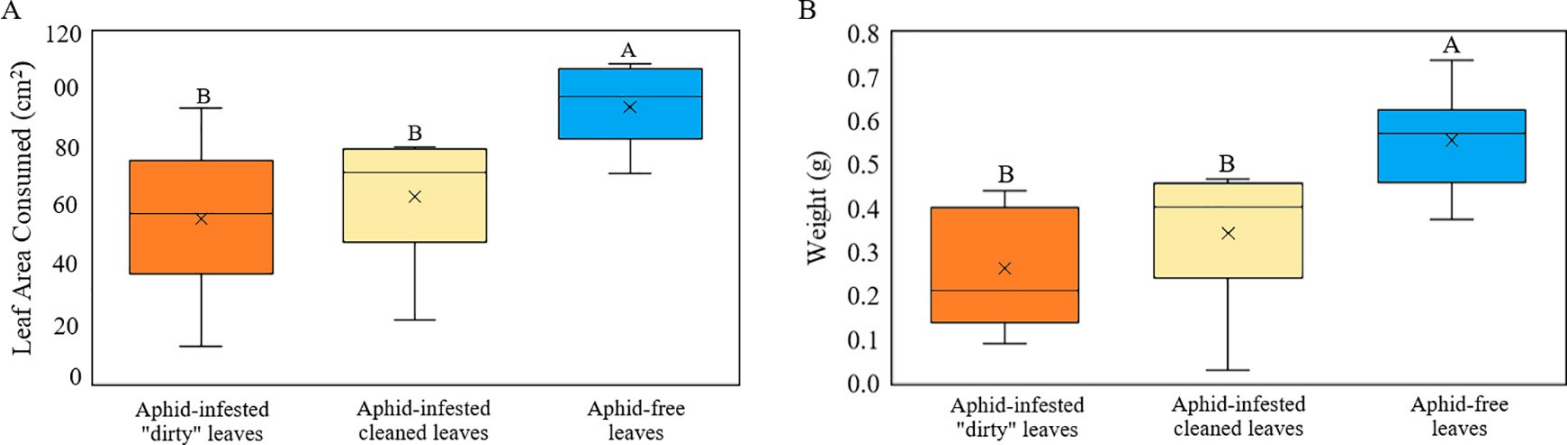
Mean (± SE) leaf area consumed over seven days (A) and final larval weight (B) of monarch larvae fed aphid-free milkweed leaves, aphid-infested “dirty” leaves, or aphid-infested cleaned leaves. Different letters over bars indicates a statistical difference between aphid infestation categories (least-squares means; A: F_2,24_ = 8.27, P = 0.001, B: F_2,24_ = 10.27, P < 0.001).

Monarch larvae fed leaves from aphid-free milkweed consumed significantly more leaf material than larvae fed leaves from either aphid-infested treatment (Fig 2, F_2,24_ = 8.27, P = 0.001). Larvae that fed on aphid-free leaves ate an average of 94.3 cm^2^ (± 4.44), with a range of 71.7–109.1 cm^2^, nearly twice the leaf material consumed by larvae fed aphid-infested “dirty” (56.1 cm^2^ ± 8.52 with a range of 12.4–93.9 cm^2^) and aphid-infested “cleaned” (63.8 cm^2^ ± 7.47 with a range of 21.4–80.8 cm^2^) leaf treatments.

As herbivory was strongly positively correlated with weight (DF = 24, R^2^ = 0.79, P < 0.001), monarch larvae fed aphid-free milkweed leaves weighed approximately twice as much (0.56 g ± 0.04, with a range of 0.37–0.74 g) as larvae fed leaves from either aphid-infested treatment (Fig 2B, F_2,24_ = 10.27, P < 0.001). Larvae that fed on aphid-infested “dirty” leaves weighed the least, with an average of 0.26 g (± 0.05) and a range of 0.09–0.44 g. Larvae fed aphid-infested “cleaned” leaves weighed an average of 0.34 g (± 0.05), with a range of 0.03–0.47g.

All larvae that fed on aphid-free milkweed survived and reached fifth (final) instar by the end of the experiment. Two larvae (22%) that fed on aphid-infested “dirty” leaves died before the end of the experiment (one third instar and one fourth instar), and three additional larvae (33%) only reached fourth instar by the end of the experiment. No larvae in the aphid-infested “cleaned” leaves treatment died, but two (22%) only reached fourth instar by the end of the experiment.

### Milkweed characteristics

Latex exudation after 30 s was no different between aphid-free and aphid-infested plants (P-value = 0.60). Aphid-free plants averaged 0.080 g (± 0.0007) latex while aphid-infested plants averaged 0.0798 g (± 0.0012) latex. Dry leaf weight per plant was nearly twice as much for aphid-free plants (DF = 16.19, T = -8.90, P < 0.001), averaging 7.74 g (± 0.34) total dry leaf weight, compared to aphid-infested plants averaging 3.92 g (± 0.26). This was reflected by a notable size difference between aphid-free and aphid-infested plants used for milkweed characteristics analysis, the latter of which were shorter and had smaller leaves. Although we could not statistically compare them, leaf nitrogen and crude protein content followed the same trend as leaf biomass, with pooled aphid-free leaves having 5.24% nitrogen and 32.75% crude protein on a dry matter basis and aphid-infested leaves having 3.76% nitrogen and 23.51% crude protein.

## Discussion

Our results demonstrate that high-density oleander aphid infestations, a common occurrence in ornamental milkweed gardens, have substantial negative effects on monarchs by reducing oviposition, larval weight, and larval leaf consumption by nearly 50%. Aphid infestations cause physical changes (e.g., clustering of aphids on undersides of leaves, buildup of honeydew and sooty mold) in addition to inducing chemical defenses, primarily cardenolides, in milkweed. Many factors influence cardenolide concentrations in milkweed, including temperature [26] and herbivory [39, 55], and plant response to each varies by milkweed species [26, 28, 29]. Importantly, herbivory on highly defended milkweed species like tropical milkweed tends to cause more pronounced effects on induced chemical defenses compared to its lower-cardenolide congeners [39, 42]. This may help explain the difference in our results from previous studies that used other milkweed species. Specifically, Ali and Agrawal [39] observed faster growth in monarch larvae fed aphid-infested *A*. *syriaca* and that aphid feeding reduced cardenolide levels in *A*. *syriaca*, which likely benefitted monarch larvae. Given that high density aphid infestations induce elevated cardenolide concentrations in the already highly-defended tropical milkweed [40], our findings likely differ because they reflect the consequence of very high cardenolide levels for larval survival and growth [26, 45]. All aphid-infested plants in our experiments had high-density infestations; therefore, these plants presumably experienced aphid-induced cardenolide increases along with sooty mold, honeydew, and aphid carcass accumulation on the leaves. Although we cannot determine whether reduced oviposition, larval feeding, or larval weight was caused by these physical changes to the plant or induced plant defenses, we can infer based on previous research [39, 46] and our findings that both likely played a role.

Oleander aphid infestations can also affect monarchs by reducing host plant quality via decreased nitrogen concentrations [55]. Lepidopterans, including monarchs, typically compensate for decreased nitrogen by increasing herbivory [56], but we observed the opposite behavior. Although our aphid-infested plants had less nitrogen, monarch larvae consumed significantly less aphid-infested leaf material than monarchs fed aphid-free plants. This suggests that induced chemical defenses or physical changes to the plant caused by aphid infestation may have inhibited compensatory feeding. As a result, larvae fed aphid-infested leaves were significantly smaller than larvae fed aphid-free plants and would likely develop into smaller adults, provided they successfully completed metamorphosis. Although we did not explicitly test effects of aphid infestation on monarch larval development rate or survival, we did observe clear differences in both between larvae in the aphid-infested and aphid-free milkweed treatments. For example, due to mortality or delayed development rate, 56% of larvae feeding on aphid-infested “dirty” milkweed did not reach final instar after seven days whereas all larvae feeding on aphid-free milkweed did. Our results likely provide a conservative assessment of plant-mediated effects on monarchs since neonates and earlier instar larvae would likely be more susceptible to plant defenses or reduced plant quality than the third instar larvae we inoculated plants with.

Taken together, the effects of high-density aphid infestations on monarchs may have significant implications for insect conservation efforts in urbanized landscapes. These results suggest that pest-ridden tropical milkweed provides reduced conservation value for monarchs as it recruits fewer monarch eggs, and those eggs will hatch into larvae with worse developmental outcomes. Although the largest swaths of milkweed plantings in North America are composed of species that may have less toxic effects than we observed with tropical milkweed, these occur predominantly in rural or agricultural lands [57]. Participation in pollinator conservation by U.S. residents, over 80% of whom reside in urban areas [58], is booming, with over 41,000 Monarch Waystation gardens registered with Monarch Watch [59] and well over 1,000,000 pollinator gardens registered as part of the National Pollinator Garden Network as of 2022 [60]. In the southern U.S., tropical milkweed is the predominant milkweed species in these spaces. Our results provide further justification for discouraging the use of tropical milkweed in urban and residential gardens due to the ubiquity of oleander aphids in these settings. Our findings also highlight the importance of suppressing aphids on tropical milkweed and, more broadly, suppressing pests on larval hosts of other lepidopteran species. This is especially relevant for highly defended plant taxa and plantings in urban landscapes where public conservation efforts are most prevalent and publicized, but where outbreaks of sap-feeding herbivores and other key arthropod pests are also commonplace and difficult to manage [6].

Three approaches to mitigating the impacts of such pervasive urban plant pests are to use plant species that are less susceptible to key pests, use plants that have less toxic secondary plant defenses, or to employ strategies that suppress aphids without negatively affecting monarchs. When conserving herbivorous insects like Lepidoptera with specific host plants, plant choice may be restricted to a relatively small pool of closely related species [61], and pest susceptibility may be high on all commercially available species or cultivars. For example, of the 35 federally listed endangered or threatened butterflies and moths, 27 have only one or two known larval host plant species [62]. Since many of these larval host plants are uncommon native species or not widely commercially available, their pest susceptibility in urban landscapes may be poorly understood and no viable alternative hosts may be available. The most common approach to aphid suppression is insecticide application, which includes a wide array of products that could have a broad spectrum of negative impacts. As pesticide use, along with habitat loss, is a major contributor to pollinator and biodiversity declines [63, 64], insecticides beyond insecticidal soaps are not currently a sustainable approach to aphid suppression on larval host plants. Although the authors did not measure aphid density or suppression, Nestle et al. [65] found that increasing floral richness in milkweed gardens increased local predatory insect abundance, which could help regulate oleander aphid densities and mitigate plant-mediated effects on monarchs. Leveraging natural biotic or abiotic regulators of aphid populations on milkweed should be investigated. Finally, although we used a non-native, highly defended milkweed species, commercial availability is a driver of consumer planting decisions [14], and tropical milkweed is well established in nursery production and trade. Using an alternative milkweed species (e.g., *Asclepias incarnata*) may reduce the effects of induced plant defenses [26–29], but oleander aphids also commonly reach high densities on these less toxic milkweed species [36]. Therefore, increasing the production and availability of alternative milkweed species may help mitigate our observed plant-mediated negative effects of oleander aphids, but would not eliminate potential effects of associated pest management inputs targeting aphid outbreaks. Further, the strategies described above do not offset the implications of our findings given the dominant market share currently occupied by tropical milkweed in nursery production and urban gardens.

This study identifies a potential widespread and under recognized impediment to monarch conservation efforts throughout the southern U.S. and sheds light on a need for providing high-quality, pest-free milkweed to support monarch conservation efforts. We also demonstrate a current void in integrated pest management tactics for ornamental plants that are larval hosts for insects of conservation concern. As public interest and effort in biodiversity conservation continues to grow, nursery production of host plants will likely follow. Thus, the relevance of pests and their management on wildlife-friendly ornamentals to biodiversity conservation will also increase [66]. Although it is well supported that some insect herbivores outbreak on urban plants [7], this response can vary by insect life histories and local landscape characteristics [67]. Improving our understanding of how urban landscape characteristics influence multitrophic interactions may help inform future management strategies that suppress pests and conserve beneficial Lepidoptera on host plants in situ without the use of insecticides that may introduce additional risk factors. Using insecticides when managing plants for conservation purposes is generally discouraged, but insecticides are currently some of the few tools that effectively suppress pests and potentially preserve the conservation value of these plants in urban settings. Given that many insecticides used to control key pests are also toxic or of unknown toxicity to insects of conservation concern like non-pest Lepidoptera species [68, 69], future research should address the potential conflicts and synergies between conservation efforts, ornamental plant selection, and pest management to develop best management practices for insect conservation in ornamental plantings. Such work could have widespread implications for the production, selection, and maintenance of conservation plantings in urbanized landscapes.

## Supporting information

S1 File This file contains descriptions of the metadata and R code and results for all statistical analyses. This file is also freely available along with the raw data file in the University of Florida Institutional Repository, which can be accessed at https://original-ufdc.uflib.ufl.edu/IR00011985/00001.
(DOCX)
