# Correction: Aphid infestations reduce monarch butterfly colonization, herbivory, and growth on ornamental milkweed

**DOI:** 10.1371/journal.pone.0317528

**Published:** 2025-01-09

**Authors:** Bernadette M. Mach, William Long, Jaret C. Daniels, Adam G. Dale

After publication of this article [1], concerns were raised by a reader about the methodology, the interpretation of the results, and the validity of the conclusions. After comprehensive correspondence with the authors and assessment by a member of our Editorial Board, the article’s overall results and conclusions are upheld, but some areas that needed clarification were identified.

Here, the authors provide additional information to clarify the following areas of expressed concern.

1. The declining monarch population referenced and studied.

In the introduction, the authors refer to the size of the overwintering migratory population, not the summer breeding population, as a measure of the monarch population [2, 3, 4, 5, 6]. The size of the overwintering migratory population is the standard measure used as an indicator of monarch population size by conservation groups and governmental agencies worldwide [4, 5, 6, 7, 8]. This measure is used because it is the most accurate assessment of the size of the (successful) migratory population at a single, critical timepoint in their migratory life history. The eastern migratory phenomenon relies on enough adult monarchs successfully overwintering to repopulate along the migratory route the following spring. Furthermore, the past 30 years have seen remarkably small overwintering populations, including this past winter in which overwintering populations occupied a mere 0.9 hectares [7]. Such small numbers of overwintering adults are incredibly vulnerable to further habitat loss or natural disasters such as landslides or winter storms, hence the emphasis on the overwintering migratory population as a marker for overall eastern monarch populations.

2. The use of the term, ornamental milkweed.

The authors recognize that the use of “ornamental milkweed” in the title may create confusion with their use of the term in other parts of the manuscript. In the title, they are referring to their study results, which focused solely on tropical milkweed, *Asclepias curassavica*. Their use of the term, “ornamental milkweed,” throughout the manuscript text refers to any milkweed species (native or nonnative) planted in a garden or managed landscape for aesthetic and/or conservation purposes.

3. The use of “conservation value” of ornamental milkweed.

The authors use the term “conservation value” in their paper to refer to the ability of any ornamental milkweed plant to support monarch larvae throughout their life cycle, particularly in the context of often depauperate urban landscapes.

4. The methods information about the monarch colony used for experiments (genetic diversity and source of founding).

The published manuscript omitted important details regarding the establishment and maintenance of the monarch colony used for experiments. The colony was founded from a mixture of wild-collected stock and stock from a local butterfly farm colony. The colony was then regularly supplemented every few weeks with wild-caught breeding monarchs from the local area in Gainesville, Florida, USA. For the lab-reared colony, 2–15 wild-caught butterflies were added to each generation to ensure that fresh wild-sourced genetics were constantly being re-introduced into the colony.

5. The potential impact of *Ophryocystis elektroscirrha* infection on the interpretation of the results.

The authors chose to not test monarchs for the protozoan parasite, *Ophryocystis elektroscirrha* (OE), during their experiments because OE infection is very common throughout monarch populations in North America, and while it can have effects apparent in adults, such as wing deformities, mortality and reduced size, the authors are not aware of any literature documenting effects at the larval stage. It is also not possible to detect infection until the adult butterfly stage. Given that a) there are no known effects at their focal life stage, b) infection would be impossible to detect at their focal life stage, and c) infection is naturally occurring in the wild population at a high level, the authors opted to not test for OE. In addition, assuming that OE was present in their colony, their randomized blocked experimental design specifically accounted for any unexplained variability in their experiment potentially introduced by OE infection. Therefore, OE prevalence in their monarch population and the potential interaction between OE infection and severity of aphid impacts to monarchs, while interesting, is outside the scope of their study and should not have influenced the results that they reported. The authors still observed an effect of oleander aphid infestations on monarch oviposition and larval performance.

6. The importance of conservation efforts that focus on discouraging use of tropical milkweed vs introducing pest control.

The authors are not suggesting the use of insecticides to suppress aphids on milkweed. Further, the authors are not recommending the use of tropical milkweed as a component of monarch butterfly conservation. Their study found that oleander aphid infestations on tropical milkweed negatively affect monarch larval growth, herbivory, and adult oviposition. These negative effects could have repercussions on the larger goal of conserving monarch butterfly populations, especially given the frequency of tropical milkweed plantings in urban landscapes which are already more prone to frequent and severe pest outbreaks. While insecticides are commonly used to manage pests like aphids, this course of action is not advised for larval host plants like milkweed. The authors indicate another recent study from their lab that provides evidence that even reduced-risk or low impact insecticides used during plant production can still negatively affect monarch larvae when used to suppress oleander aphids on milkweed [1]. Hence, the authors emphasize in the discussion that alternative methods are needed, namely by using plant species that are less susceptible to key pests like aphids, using plants that have less toxic secondary plant defenses, or employing other strategies that suppress aphids without negatively affecting monarchs (i.e. by physical removal or enhancement of natural enemies). Because tropical milkweed is particularly susceptible to aphid infestations and has very toxic secondary plant defenses, the authors argue that their study adds to the growing evidence discouraging the use of tropical milkweed. The authors note they chose to study tropical milkweed not because they want to promote its use, but because it is incredibly relevant as the most widely commercially available milkweed in the southeastern United States. Thus, the authors consider it imperative to understand how this ubiquitous pest can affect monarchs on this ubiquitous milkweed species because this very scenario (monarchs feeding and ovipositing on aphid-infested tropical milkweed) is likely playing out in thousands of yards, parks, and gardens every year in the United States. Finally, their study highlights an important void, beyond the scope of the monarch-milkweed system, in current integrated pest management tactics for ornamental plants that are larval hosts for beneficial insects. Their findings also highlight yet another a disconnect between the production/demand for tropical milkweed versus the mounting body of evidence discouraging its use in the US.
