# Supplementary material for: Aphid infestations reduce monarch butterfly colonization, herbivory, and growth on ornamental milkweed
Source: PLoS One. 2023 Jul 26;18(7):e0288407. doi: 10.1371/journal.pone.0288407 (PMC10370756; doi:10.1371/journal.pone.0288407)
Supplement: S1 File — This file is also freely available along with the raw data file in the University of Florida Institutional Repository, which can be accessed at https://original-ufdc.uflib.ufl.edu/IR00011985/00001. (DOCX) [file pone.0288407.s001.docx]

CODE FOR STATISTICAL ANALYSES

*Oviposition Study*

# OVI_DATA – xlxs file containing the columns TRT, DATE, PAIR, and EGGS

# TRT – treatment, either NOA (no aphids) or HIA (high aphid pressure)

# DATE – date of the trial, either JUN (June) or SEP (September)

# PAIR – monarch pair/cage

# EGGS – number of eggs collected

# CONDUCT GLMER

require(lme4)

OVIPOSIT <- glmer(EGGS ~ TRT + (1|PAIR) + DATE, family = poisson, data = OVI_DATA)

summary(OVIPOSIT)

# OUTPUT

Generalized linear mixed model fit by maximum likelihood (Laplace Approximation) ['glmerMod']

Family: poisson ( log )

Formula: EGGS ~ TRT + (1 | PAIR) + DATE

Data: OVI_DATA

AIC BIC logLik deviance df.resid

238.6 242.2 -115.3 230.6 14

Scaled residuals:

Min 1Q Median 3Q Max

-4.0223 -1.6224 -0.4678 1.6654 4.1652

Random effects:

Groups Name Variance Std.Dev.

PAIR (Intercept) 1.005 1.003

Number of obs: 18, groups: PAIR, 9

Fixed effects:

Estimate Std. Error z value Pr(>|z|)

(Intercept) 2.60126 0.45839 5.675 1.39e-08 ***

TRTNOA 1.19306 0.08605 13.864 < 2e-16 ***

DATESEP -0.07830 0.68262 -0.115 0.909

---

Signif. codes: 0 ‘***’ 0.001 ‘**’ 0.01 ‘*’ 0.05 ‘.’ 0.1 ‘ ’ 1

Correlation of Fixed Effects:

(Intr) TRTLOA

TRTLOA -0.144

DATESEP -0.657 0.000

*Larval Feeding Study*

# FEEDINGASSAY – xlxs file containing the columns TREAT and LEAF

# TREAT – treatment, either HIA (high aphids "dirty" leaves), HIC (high aphids

cleaned leaves), or LOC (low/no aphids clean leaves)

# LEAF – leaf area consumed

# WEIGHT – final larval weight

# Compute ANOVA for total leaf area consumed

# CODE

aov3(LEAF ~ TREAT, FEEDINGASSAY)

# OUTPUT

Response : LEAF

Df Sum Sq Mean Sq F value Pr(>F)

MODEL 2 7346.8 3673.4 8.2677 0.001856 **

TREAT 2 7346.8 3673.4 8.2677 0.001856 **

RESIDUALS 24 10663.4 444.3

CORRECTED TOTAL 26 18010.2

# Separate means using LSM

# CODE

LSM(LEAF ~ TREAT, FEEDINGASSAY, conf.level=0.95, adj="lsd",

hideNonEst=TRUE, PLOT=TRUE)

# OUTPUT

Group LSmean LowerCL UpperCL SE Df

LOC A 94.32144 79.82008 108.8228 7.026199 24

HIC B 63.76833 49.26697 78.2697 7.026199 24

HIA B 56.14633 41.64497 70.6477 7.026199 24

# ---

# Compute ANOVA for final larval weight

# CODE

aov3(WEIGHT ~ TREAT, FEEDINGASSAY)

# OUTPUT

Response : WEIGHT

Df Sum Sq Mean Sq F value Pr(>F)

MODEL 2 0.37843 0.189216 10.266 0.0007094 ***

TREAT 2 0.37843 0.189216 10.266 0.0007094 ***

RESIDUALS 22 0.40551 0.018432

CORRECTED TOTAL 24 0.78394

# Separate means using LSM

# CODE

LSM(WEIGHT ~ TREAT, FEEDINGASSAY, conf.level=0.95, adj="lsd",

hideNonEst=TRUE, PLOT=TRUE)

# OUTPUT

Group LSmean LowerCL UpperCL SE Df

LOC A 0.5551111 0.4612580 0.6489642 0.04525499 22

HIC B 0.3425556 0.2487024 0.4364087 0.04525499 22

HIA B 0.2624286 0.1560091 0.3688480 0.05131434 22
